# A rare asymptomatic metallic intraocular foreign body retained in the anterior chamber for 15 years: A case report

**DOI:** 10.1097/MD.0000000000026470

**Published:** 2021-06-25

**Authors:** Na He, Zhigang Lv

**Affiliations:** Department of Ophthalmology, Affiliated Jinhua Hospital, Zhejiang University School of Medicine, Jinhua, China.

**Keywords:** case report, electroretinogram, intraocular foreign body, ocular siderosis

## Abstract

**Rationale:**

Intraocular foreign bodies (IOFBs) are common in ocular injuries, but asymptomatic metallic IOFBs retained in the anterior chamber for years are rare.

**Patient concerns:**

A 31-year-old female presented with blurred vision in her right eye after lumbar magnetic resonance imaging. Her best-corrected vision acuity was 0.6 in the right eye and 1.0 in the left eye. Slit-lamp examination revealed a brown granular foreign body in the anterior chamber and pigmentation of the limbus. Lens and retina examination indicated ocular siderosis. Corneal endothelioscopy revealed decreased endothelial cell density. A detailed history showed ocular globe injury 15 years earlier.

**Diagnoses:**

Anterior chamber IOFB with ocular siderosis.

**Interventions:**

Anterior chamber foreign body removal was performed with appropriate incision and forceps.

**Outcomes:**

The anterior chamber IOFB was successfully removed and examined as a magnetic metal foreign body. The best-corrected vision acuity was 1.0 at 1 day postoperatively. An abnormal electroretinogram with a 12% decrease in the “b” wave and a 91% decrease in the “a” wave was observed 3 months postoperatively. There were no intraoperative or postoperative complications during a 3-month follow-up.

**Lessons:**

Eye trauma should be examined carefully to exclude IOFBs. Asymptomatic anterior chamber foreign bodies can also cause corneal endothelial injury and ocular siderosis. Careful examination and timely management are needed in such cases.

## 1 Introduction

Intraocular foreign bodies (IOFBs) are common in penetrating ocular injuries and can often cause serious visual loss.^[1]^ The incidence of IOFB in open globe injury is 18% to 41%.^[1,2]^ IOFBs can result in direct mechanical damage and subsequent metallosis, such as ocular siderosis (OS) and chalcosis^[2]^; however, the most serious complication is infectious endophthalmitis.

Ruptured eyeball repair and immediate IOFB removal are always recommended to avoid endophthalmitis.^[3]^ Long-term retained or misdiagnosed IOFBs may occur when patients do not pay attention or do not have access to medical care, but most patients will suffer from partial vision impairment. Asymptomatic metallic IOFBs retained in the anterior chamber for years are rare. Here, we report a 31-year-old female with an asymptomatic metallic anterior chamber foreign body that was discovered 15 years after undiagnosed penetrating ocular trauma. We successfully removed the foreign body, but the patient still had irreversible corneal endothelial injury and early OS.

## 2 Case report

A 31-year-old Chinese female presented to the ophthalmic clinic of our hospital and complained of persistent blurred vision in her right eye for 1 day immediately after a lumbar spine magnetic resonance imaging (MRI). As a routine procedure, she was asked before the scan if she had any contraindications to the MRI scan. At that time, she denied any metallic foreign body or surgical devices. After the MRI scan, she noticed blurred vision in her right eye immediately, but she did not take it seriously until her symptoms persisted for one day.

**Figure 1:**
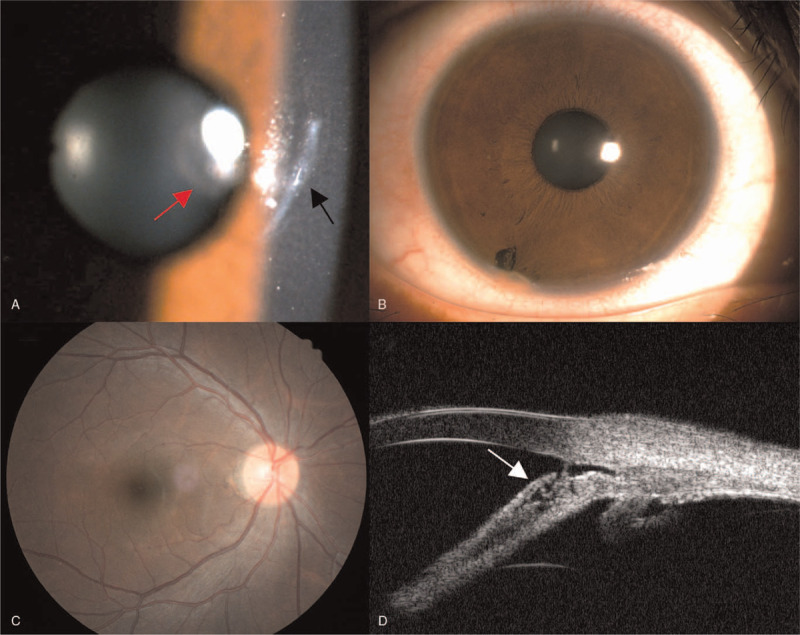
A: The slit-lamp image of the anterior segment on initial examination showed a 2 mm paracentral corneal linear scar (black arrow) and brownish spots on the anterior lens capsule (red arrow); B: A metal particle was embedded in the iris root at 7 o’clock with the pigmentation of the adjacent limbus; C: The fundus examination showed no clinically significant abnormalities; D: Ultrasound biomicroscopy revealed the foreign body (white arrow) was lodged near the anterior chamber angle and wrapped by the adjacent tissues.

On initial examination, the uncorrected visual acuity in the right eye was 20/80, the manifest refraction was −1.00 DS–1.50 DC×160, the best-corrected vision acuity (BCVA) was 20/40, and the intraocular pressure was 11.6 mm Hg. Slit-lamp examination of the right eye showed a 2 mm linear corneal scar on the nasal side of the pupil (Fig. 1A) and a metal particle embedded in the iris root at 7 o’clock with the pigmentation of the adjacent limbus (Fig. 1B). Brownish spots were observed on the anterior lens capsule at the pupil area (Fig. 1A). No corneal edema but mild anterior chamber inflammation was observed. The examination of the left eye was unremarkable. Further detailed history revealed a previous ocular injury concerning an undiagnosed IOFB 15 years earlier while her father was pounding metal against metal with a hammer. After intravenous antibiotic therapy at a local clinic, she did not feel any discomfort; thus, she was never referred to an ophthalmologist. She noticed an abnormality in the appearance of her eye in the mirror but thought it was a scar on her cornea and ignored it.

**Figure 2:**
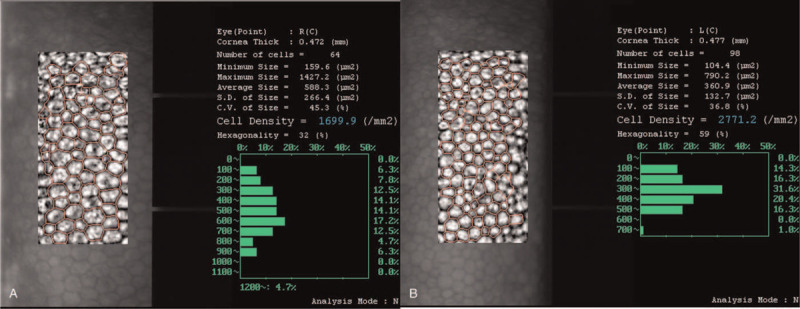
Specular microscopy of the right eye (A) revealed decreased corneal endothelial cells density (1699.9/mm^2^ vs 2771.2/mm^2^) and percentage of hexagonal cells (32% vs 59%), compared with the left eye (B).

**Figure 3:**
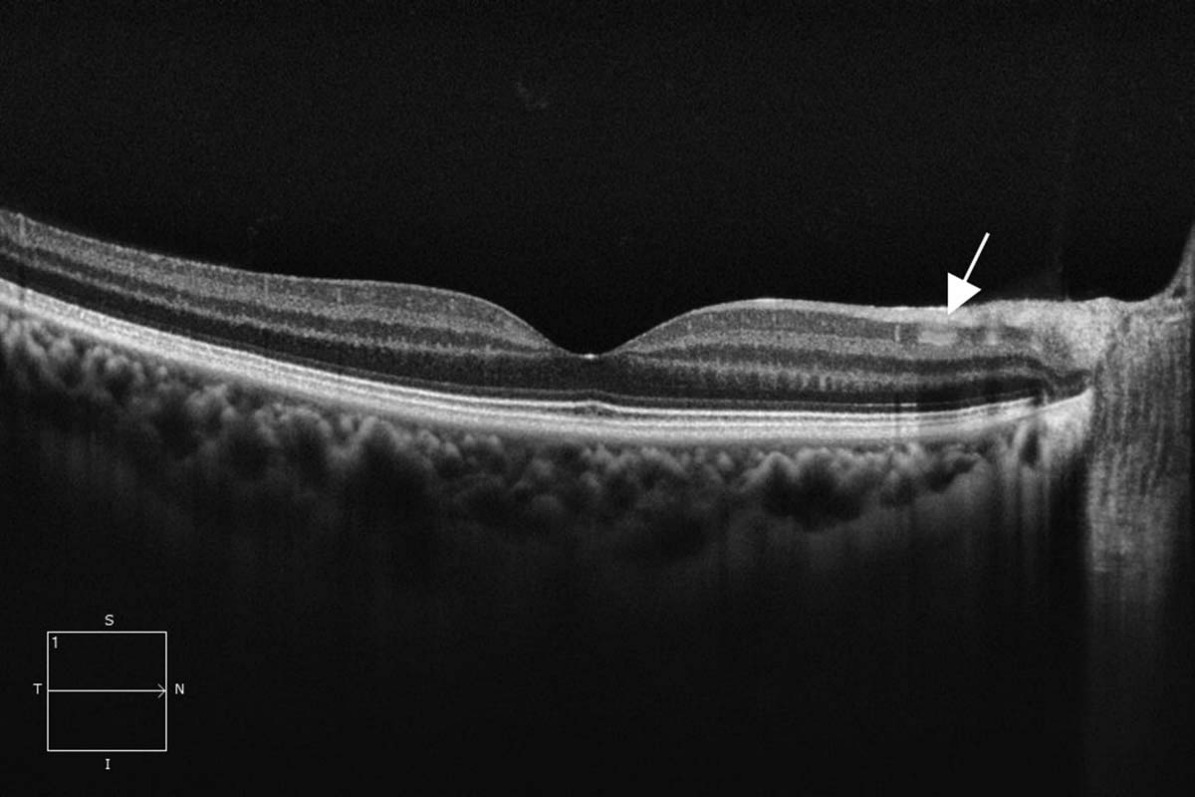
Posterior segment OCT showed degeneration of the inner retina (white arrow). OCT = optical coherence tomography.

Fundus examination and photography showed no pigmentary retinal degeneration, optic disc swelling, or hyperemia (Fig. 1C). Ultrasound biomicroscopy showed that the foreign body was lodged near the anterior chamber angle (Fig. 1D). A non-contrast computerized tomography scan of the orbits confirmed a radiopaque foreign body embedded in the anterior chamber, and no other foreign body was detected. Corneal endothelioscopy revealed decreased corneal endothelial cell density and percentage of hexagonal cells compared with the contralateral eye (Fig. 2). The posterior segment swept-source optical coherence tomography examination revealed inner retinal degeneration at the peripapillary area despite an intact and normal microstructure of the foveal region (Fig. 3). Electroretinogram (ERG) examination was not performed because of the poor toleration and cooperation of the patient at that time. Thus, she was diagnosed with anterior chamber IOFB (ACFB) and OS.

**Figure 4:**
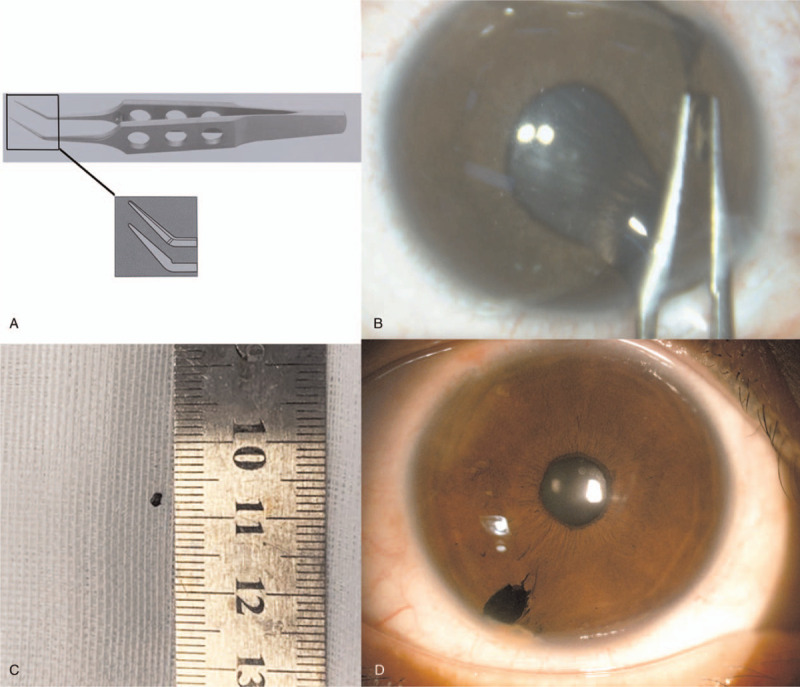
A: The tying forceps with 0.3 mm wide tips, 5 mm tying platforms, and 10 mm angled shafts from tips to bend; B: The ACFB was grasped by the tying forceps; C: The 1.5 mm × 1.0 mm × 1.0 mm extracted brown iron particle; D: The slit-lamp image showed mild anterior chamber inflammation and a small iris defect near the limbus.

**Figure 5:**
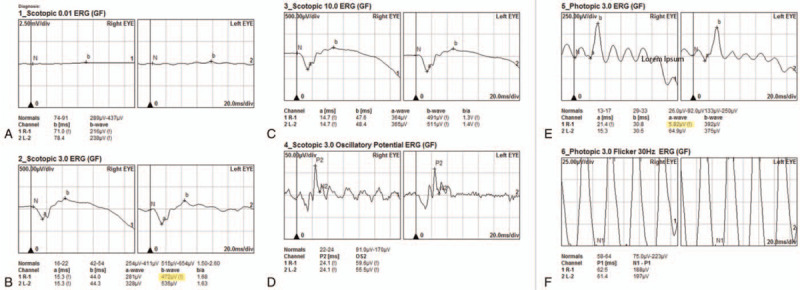
ERG three months postoperatively showed a 12% decrease in the “b” wave and a 91% decrease in the “a” wave.

IOFB extraction was advised for this patient. The surgery was performed under topical anesthesia. A limbal incision was made with a 3.0 mm slit knife at 11 o’clock. An ophthalmic viscosurgical device was refilled to stabilize the anterior chamber and protect the corneal endothelium. A pair of tying forceps with 0.3 mm wide tips, 5 mm tying platforms, and 10 mm angled shafts from tips to bend (Fig. 4A) was used to extract the IOFB. The IOFB was successfully isolated from granulation and adjacent tissues and then extracted from the eye (Fig. 4B). The ophthalmic viscosurgical device was aspirated with irrigation-aspiration. The limbal wound was sealed by hydration. The foreign body was a 0.5 mm × 0.5 mm × 1.5 mm brown iron particle (Fig. 4C). Levofloxacin eye drops (Cravit, Santen, Japan) and tobramycin/dexamethasone eye drops (Tobradex, Alcon) were applied four times a day postoperatively. The uncorrected visual acuity in the right eye was 20/40, and the manifest refraction was −1.25 DS/−2.00 DC∗160 with a BCVA of 20/20 the next day. Anterior chamber inflammation was mild, and a small iris defect near the limbus was noticed, which may account for the photophobia of the patient (Fig. 4D). No postoperative complications were observed during a 3-month follow-up. There was a 12% decrease in the “b” wave and a 91% decrease in the “a” wave in the ERG three months postoperatively (Fig. 5).

## 3 Discussion

The previously reported incidence of IOFB in the general population is 0.27%^[4]^ and ranges from 18% to 41% in open-globe injuries.^[5]^ Most posttraumatic IOFBs reside in the posterior segment (58%–88%), while the others are in the anterior chamber (10–15%) or the lens (2–8%).^[2]^ Hammering accounts for the majority (60–80%) of IOFBs, followed by power equipment or machinery (18–25%) and weapon-related injuries (19%).^[6]^ Most IOFBs can be diagnosed and treated properly if the patient seeks medical care immediately; however, some IOFBs may be missed diagnosed because the wound is very small, and some patients do not feel any pain or experience visual loss. In this patient, IOFB was misdiagnosed because she recovered after antibiotic treatment and had not consulted an ophthalmologist. Although hammering is the most common cause of intraocular foreign body trauma, it was not given enough attention from a non-ophthalmologist. When eye trauma occurs, an ophthalmologist consultation is important to exclude intraocular foreign bodies.

Adverse events caused by IOFB during MRI examinations are rarely reported. Previously a metallic IOFB was found in a 12-year-old child by brain MRI without complications.^[7]^ The foreign body was embedded in the retina. This case was not treated surgically, as no visual impairment or ocular toxicity occurred at the initial examination or during a 7-years follow-up. In another case, a 47-year-old male with an undetected metallic IOFB experienced focal hyphema after MRI scans.^[4]^ After a 15-days follow-up, the patient's vision returned to normal, the clotted blood was absorbed, and a foreign body was found embedded in the iris stroma. Therefore, they decided to manage that case nonsurgically and follow up closely for the development of the cataract progression, recurrent hyphema, secondary glaucoma, and OS. Our patient was referred to the doctor because of blurred vision caused by MRI and IOFB removal was performed since OS developed. These cases highlight the importance to rule out any possibility of a magnetic foreign body caused by trauma before the MRI scan.

Retained IOFBs can cause a variety of complications. The most serious is endophthalmitis. According to previous references, the incidence of endophthalmitis associated with IOFB after open-globe injuries ranges from 4.7% to 13.5%.^[2,5,8]^ Adequate antibiotic administration and prompt foreign body removal can reduce the risk of endophthalmitis. In addition, IOFBs can also result in direct mechanical damage and consequential metallosis. Iron and copper ions released by metallic foreign bodies are toxic to the eye and can induceOS, chalcosis, iris heterochromia, secondary glaucoma, and eventually optic atrophy. A retained ACFB can cause severe corneal endothelium injury, corneal edema, or even corneal decompensation because of long-term friction^[9]^ and can also stimulate pigmentation and uveitis. The present case showed corneal endothelium injury and OS 15 years after the initial open-globe injuries. The ACFB was wrapped by granulation and adjacent iris tissue; thus, it could not move freely with the patient's movement. For this reason, there was no corneal endothelium decompensation despite the long-term retention of ACFB; however, continuous release of iron ions resulted in OS. Optical coherence tomography results of the retina with OS were not frequently reported. In our case, the inner retina showed degeneration, which was consistent with a previous case.^[10]^

ACFB requires comprehensive evaluation and prompt management to avoid sight-threatening complications. The removal of ACFB was obligatory in the present case because of OS. Various forceps and rare earth intraocular magnets are the most common instruments to extract IOFBs.^[2]^ In the present case, we used tying forceps considering that its bent angle facilitated the operation from the superior limbus incision, avoiding the influence of the eyebrow arch. Besides In addition, typing forceps can provide an effective gripping platform to grasp the wrapped foreign body, and the narrow tip prevents damage to the anterior angle. ACFB removal through a secondary corneal or limbal incision is recommended because of potential iatrogenic injury through the entry wound.^[11]^ The conventional approach is to make a short and steep tunnel incision close to the ACFB. Here, we made a 3 mm corneal incision 90 degrees away from the ACFB at 10 o’clock to avoid iris prolapse and anterior angle injury. As a result of long-term chronic inflammatory stimulation, the ACFB was encapsulated very tightly. We gently separated the ACFB and the encapsulated tissues that might contain metallic ions and removed them from the corneal incision. After a well-designed operation, we performed the procedure successfully and had a BCVA of 20/20 with no complications during the 3-month follow-up.

A 12% decrease in the “b” wave and a 91% decrease in the “a” wave in the full field-ERG (ffERG) was observed in the patient's right eye 3 months postoperatively. ffERG is the gold standard for detecting retinal damage, such as toxic retinopathy and cone-rod dysfunction caused by iron IOFB, because it can reveal retinal dysfunction before any pathological changes.^[12]^ Casini et al^[13]^ mentioned that OS could be reversible and that ffERG amplitudes may increase after IOFB removal. We did not perform ffERG before the surgery; however, the postoperative ffERG showed decreased “b” and “a” waves 3 months after IOFB removal. According to the classification method of Knave^[13]^ and the results of the fundus examination, we thought this patient was at type 1 or subnormal stage. Although iron retinal toxicity is reversible in the early stage of OS, some patients still experience OS progression after IOFB removal.^[10]^ Thus, patient follow-up is necessary after surgery.

## 4 Conclusion

Eye trauma should be examined carefully to exclude intraocular foreign bodies. Asymptomatic anterior chamber foreign bodies may also cause potential corneal endothelium injury and OS, which should be carefully examined and extracted using appropriate surgical methods to avoid iatrogenic injury.

## Author contributions

**Supervision:** Zhigang Lv.

**Writing – original draft:** Na He.

**Writing – review & editing:** Na He, Zhigang Lv.
